# Editorial: Overlapping Phenotypes and Genetic Heterogeneity of Rare Neurodevelopmental Disorders

**DOI:** 10.3389/fneur.2021.711288

**Published:** 2021-07-22

**Authors:** Francesca Cogliati, Francesca Forzano, Silvia Russo

**Affiliations:** ^1^Research Laboratory of Medical Cytogenetics and Molecular Genetics, Istituto Auxologico Italiano, Istituto di Ricovero e Cura a Carattere Scientifico (IRCCS), Milan, Italy; ^2^Clinical Genetics Department, Guy's & St Thomas' National Health System Foundation Trust, London, United Kingdom

**Keywords:** overlapping phenotypes, pleiotropy of a single gene, microcephaly, SETBP1 gene, SETD5 gene, MRD23 syndrome, ring20, POLR3A

This Special Issue deals with the complexity of the interplay between genotype and phenotype. The collection consists of five articles, each focusing on a different, rare neurodevelopmental disorder, which exemplify: (a) overlapping phenotypes; (b) pleiotropy of individual gene and (c) heterogeneity within a single rare disorder. These articles provide an overview of the possible scenarios a geneticist could face with an intricate array of apparently similar cases, but that need a deep knowledge of clinical and biological mechanisms to achieve a diagnosis.

## Overlapping Phenotypes

Crippa et al. investigated a cohort of patients with a suspected KBG syndrome, who had already been screened for variants within *ANKRD11* (OMIM*611192), the main gene associated with this condition. The combination of omic approaches, array-CGH, and WES led to an unexpected finding of pathogenic variants in the *SETD5* (OMIM*615743) gene, already known to be involved in MRD23 syndrome and 3pter-p25 microdeletion syndrome. A refined clinical re-evaluation, taking into account mild intellectual disability, developmental delay, autistic spectrum disorder and atrioventricular septal defects enabled the researchers to recognize a significant clinical overlap between reported *ANKRD11-* and *SETD5-*mutated cases. However, differences in physical traits, as hand anomalies and some facial traits were highlighted. These phenotypic subtleties should not be overlooked in the pursuit of a proper clinical diagnosis. Crippa et al. propose that KBG and MRD23 may be considered overlapping, even distinct, syndromes. Interestingly, pathogenic variants in both *SETD5* and *ANKRD11* genes have been reported in patients with Cornelia de Lange Syndrome, another overlapping syndrome belonging to a group of cohesin-related disorders. According to the authors, the reasons for phenotypic overlap may be the functional role and interactions of these gene pathways. The two proteins, SETD5 and ANKRD11, both functionally related chromatin-associated factors and highly expressed in the brain, have been proven to interact with the histone deacetylase 3 (HDAC3), a key regulator of histone acetylation/deacetylation balance, and with each other.

Peron et al. discuss the genetic mechanisms leading to the rare occurrence of a non-supernumerary ring chromosome 20 [r(20)] as well as the differential diagnoses and overlapping phenotypes of the Ring Chromosome 20 Syndrome. The putative etiology of the two groups of r(20), the most common mosaic and the rarer non-mosaic r(20), is explored in detail, as well as the recurrence risk when a mosaic mother transmits the r(20) to the mosaic child. This rare condition is listed among the epileptic encephalopathies, with a “core phenotype” characterized by cognitive decline and behavioral problems that develop after the onset of a very distinctive epileptic phenotype characterized by the triad of refractory frontal lobe focal seizures, Non-Convulsive Status epilepticus (NCSE), and a typical EEG. Overlapping phenotypes in autoimmune diseases, psychiatric or genetic syndromic conditions are easily distinguishable from r(20) syndrome based on the EEG specificities, particularly on prolonged video-EEG monitoring. In this disorder, the pitfall is represented by not requesting an “old-fashioned” standard karyotype as a diagnostic test in the era of high-throughput technologies, in the presence of typical electro-clinical characteristics.

## Pleiotropy of A Single Gene

Leonardi et al. present three new cases carrying a pathogenetic variant in the *SETBP1* gene (OMIM 611060), whose missense variants are located in the specific degradation sequence (degron motif), or near this sequence, and are responsible, respectively, for the classical or milder form of Schinzel-Giedion Syndrome (SGS). The reported cases (two carrying an LoF variant and one a missense close to the degron motif), highlight the variable severity of the phenotype, from non-specific neurodevelopmental disorder to developmental and epileptic encephalopathy (DEE), and raise the hypothesis of pleiotropy for this gene. The apparent incomplete penetrance or extremely variable expressivity would seem to be due to protective genetic factors able to attenuate the SGS/SGS-like phenotype, a hypothesis suggested by the evidence that the missense variant, already identified in the literature in severe NDD and epileptic encephalopathy is not *de novo* but inherited from an asymptomatic parent, in whom it occurred *de novo*.

## Heterogeneity Within A Single Rare Disorder

The complex genetic etiology of the autosomal recessive primary microcephaly (MCPH), a congenital disease characterized by a reduced number of neurons in the developing neocortex, is discussed in Furukawa et al. As the diagnostic yield for MCPH ranges from 29% of cases after WES to 50% after WGS, an alternative winning strategy to address MCPH genes could be to take into consideration the cellular basis of the disorder and look for pathogenic variants in the genes controlling the timing of the neurogenic program. Fine dissection of the four key overlapping processes that affect cell cycle progression, and specifically the centrosome cycle, is given to the readers to clarify the role of the 25 OMIM proteins currently associated with MCPH. Going so deep into the analysis of the relation between the specific pathway deregulation and the onset of MCPH, the real aim of the Authors is to suggest how it may be more helpful to investigate mutations in the novel genes related to the same cellular mechanism to disclose the pathogenic variant of a specific rare disorder. To this purpose, functional studies on animal models are powerful tools that should more often be used.

The concept of pleiotropy is well-described in the case report by Jean et al. regarding a young lady affected with a form of leukodystrophy who is a compound heterozygote for pathogenic variants in the *POLR3A* gene. The phenotype also includes a very severe form of osteoporosis, a feature not often considered in patients with this diagnosis, but which is important to investigate and treat because of potentially severe complications.

## Author Contributions

All authors contributed by drafting the work, revising it critically for important intellectual content, and provided approval for publication of the content.

## Conflict of Interest

The authors declare that the research was conducted in the absence of any commercial or financial relationships that could be construed as a potential conflict of interest.

